# Supplementary material for: Molecular Identification and Expression Analysis of NOD1/2 and TBK1 in Response to Viral or Bacterial Infection in the Spotted Knifejaw (Oplegnathus punctatus)
Source: Animals (Basel). 2025 Mar 31;15(7):1006. doi: 10.3390/ani15071006 (PMC11987823; doi:10.3390/ani15071006)
Supplement: Supplementary file 1 [file animals-15-01006-s001.zip › animals-3455519-supplementary.pdf]

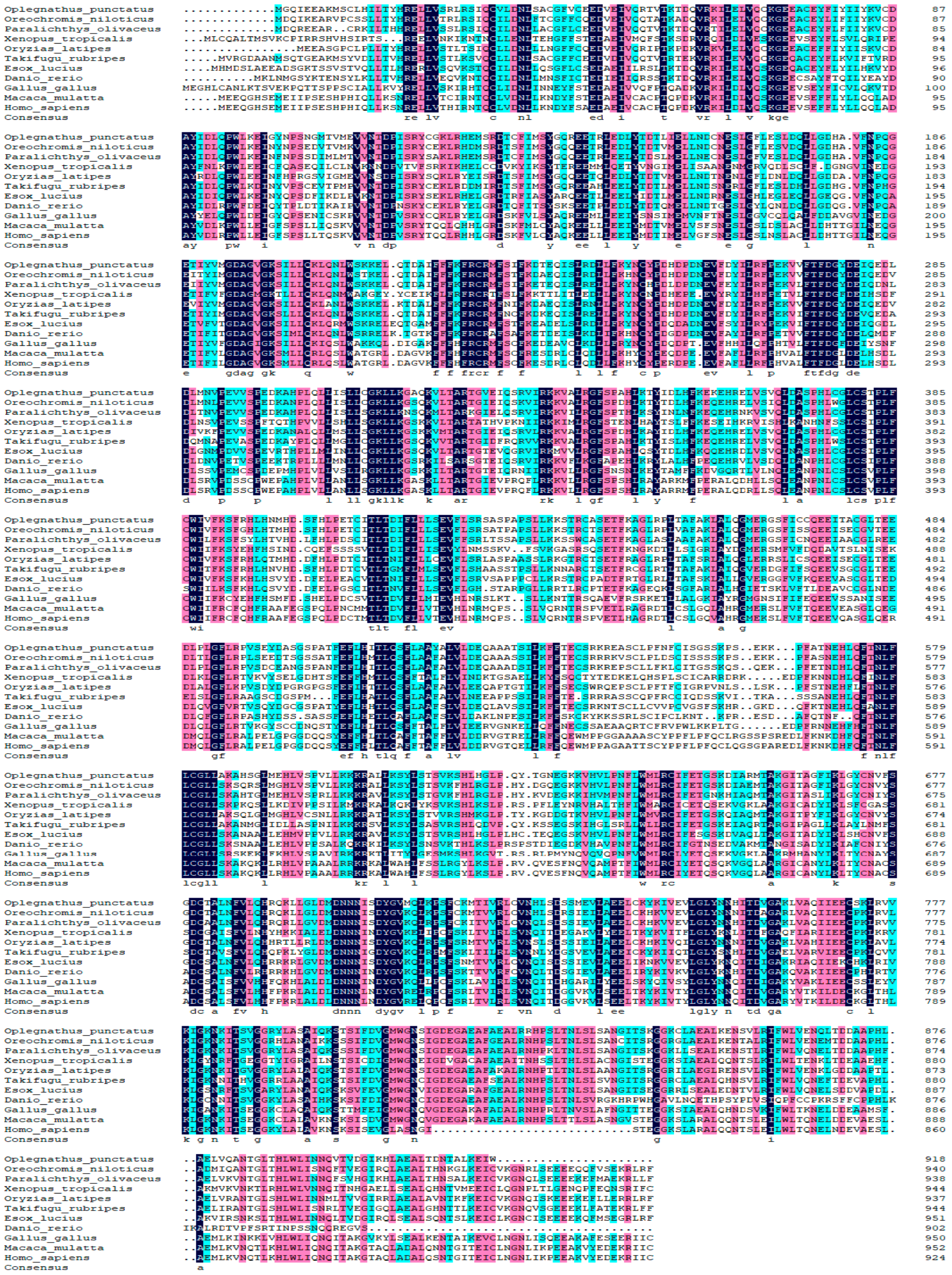

Figure S1. Multiple sequence alignment of the deduced amino acids of NOD1 among the spotted knifejaw and other species

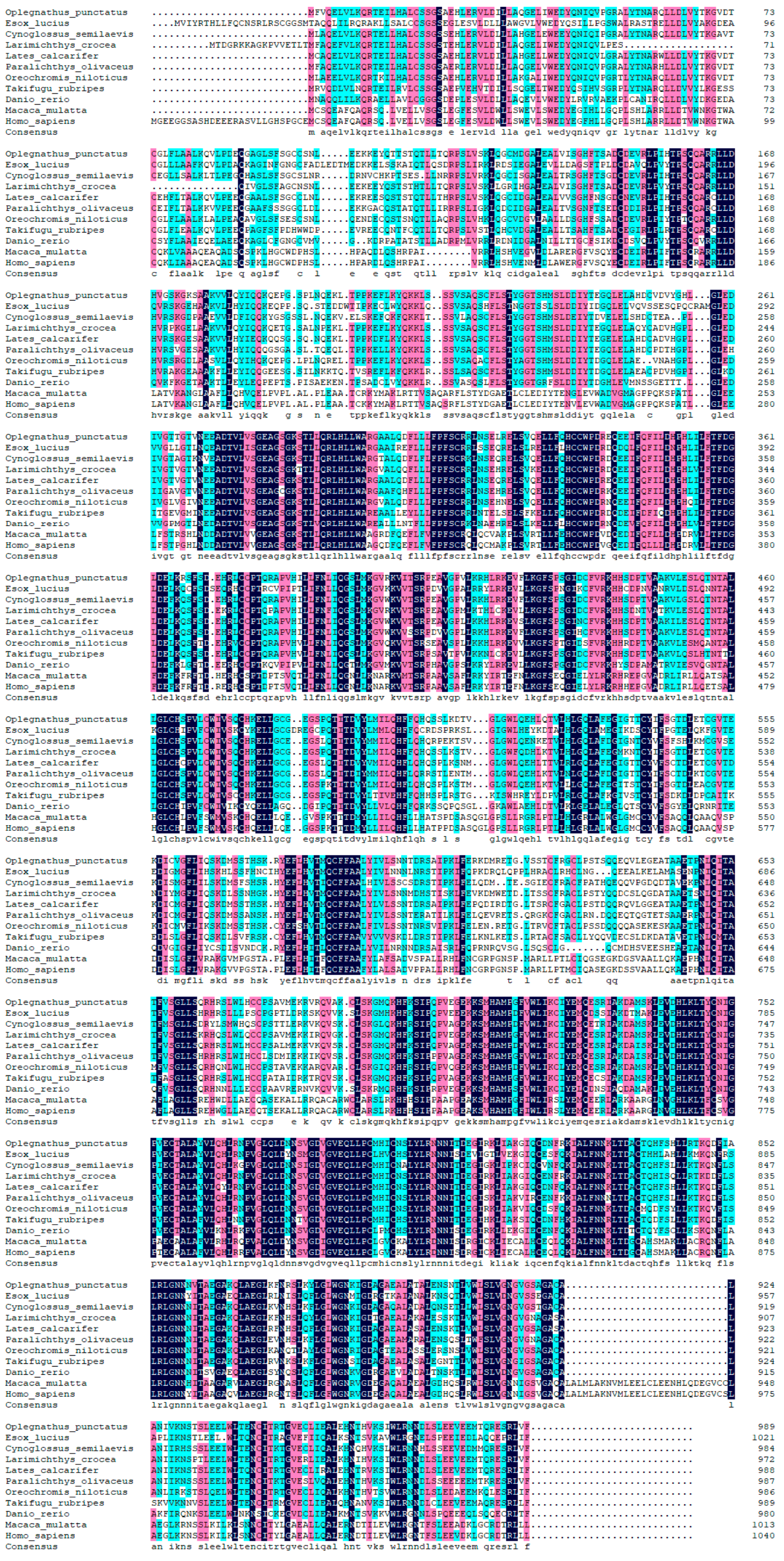

Figure S2. Multiple sequence alignment of the deduced amino acids of NOD2 from the spotted knifejaw and other species

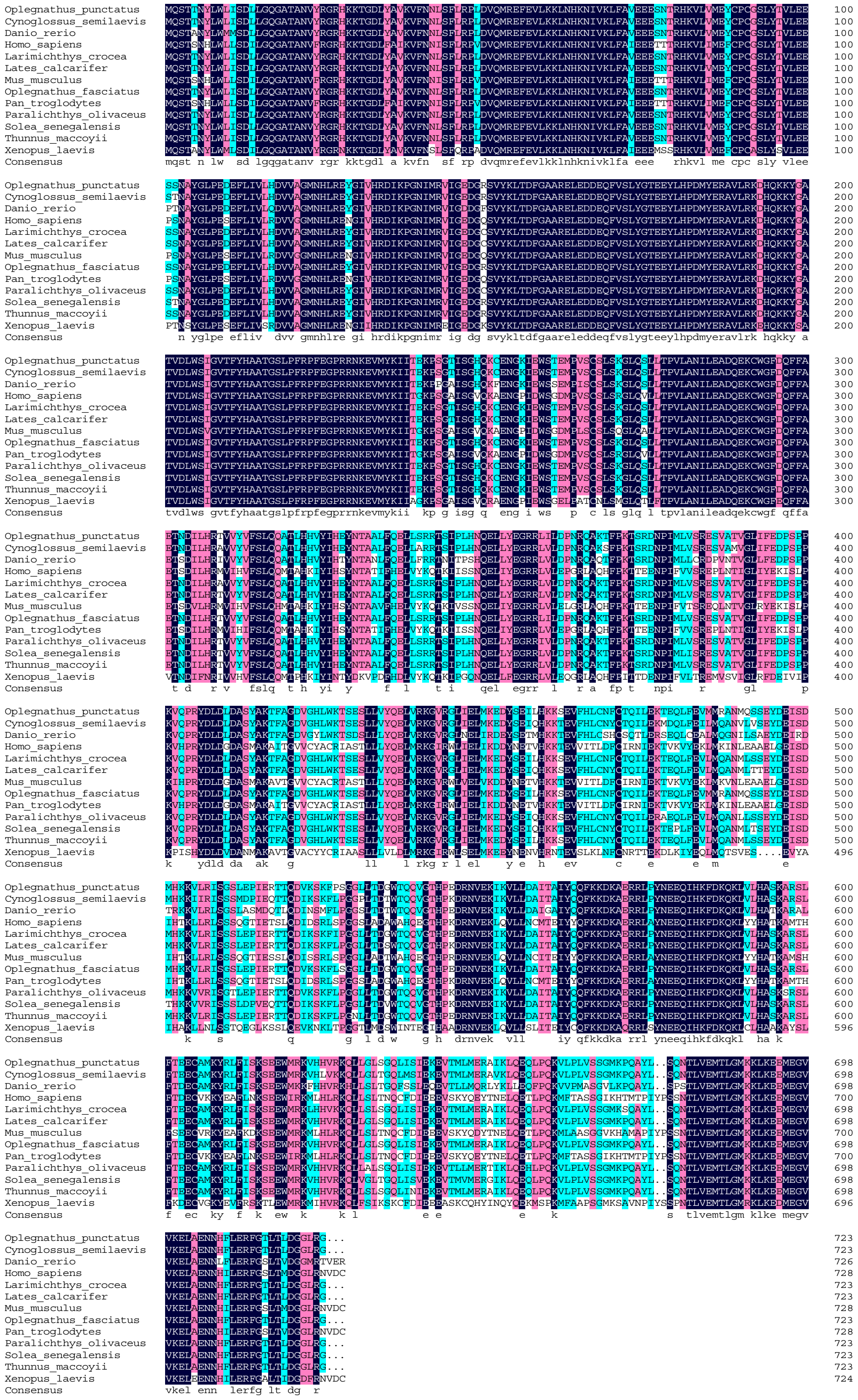

Figure S3. Multiple sequence alignment of the deduced amino acids of TBK1 from the spotted knifejaw and other species
